# Effects of methyl jasmonate on the growth and triterpenoid production of diploid and tetraploid *Centella asiatica* (L.) Urb. hairy root cultures

**DOI:** 10.1038/s41598-019-54460-z

**Published:** 2019-12-10

**Authors:** Khoa Van Nguyen, Benyakan Pongkitwitoon, Thanika Pathomwichaiwat, Unchera Viboonjun, Sompop Prathanturarug

**Affiliations:** 1 Department of Pharmaceutical Botany, Faculty of Pharmacy, Mahidol University, 447 Sri-ayuthaya Road, Bangkok, 10400 Thailand; 2 Department of Plant Science, Faculty of Science, Mahidol University, 272 Rama VI Road, Ratchathewi, Bangkok, 10400 Thailand

**Keywords:** Plant biotechnology, Secondary metabolism

## Abstract

In this study, the effects of methyl jasmonate (MeJA) on the phytomass and triterpenoid production of diploid and tetraploid *Centella asiatica* hairy roots were investigated. Hairy root cultures were obtained from diploid and induced tetraploid plants of *C. asiatica* infected by *Agrobacterium rhizogenes* strain ATCC 43057. MeJA triggered triterpenoid production in both ploidy hairy roots, whereas triterpenoids were not produced in the untreated hairy roots. Among the treatments, the 50 µM MeJA treatment yielded the maximum triterpenoid production in diploid hairy roots of 27.25 ± 0.27 µg/mg Dry weight (DW) total triterpenoid at day 21. For the tetraploid hairy root cultures, the 28th-day hairy root culture produced a maximum amount of triterpenoids of 16.29 ± 6.32 µg/mg DW in response to the 50 µM MeJA treatment, whereas the 100 µM MeJA treatment produced a similar triterpenoid amount (16.31 ± 9.24 µg/mg DW) at day 14. Moreover, in response to 50 µM MeJA, we obtained different ratios of aglycone to glycoside, i.e., 1:7 and 1:2, between the diploid and tetraploid hairy root cultures. Asiaticoside was the dominant phytochemical, followed by asiatic acid and madecassic acid. This study provides valuable information for producing triterpenoids for *C. asiatica* commercial products and preparations by using hairy root cultures.

## Introduction

*Centella asiatica* (L.) Urb. (Apiaceae) is a well-known medicinal plant in tropical and subtropical regions. The plant is a main ingredient of Ayurvedic preparations (India), traditional Chinese medicine (China), and commercial drugs in the European market^1,2^. Madecassoside, asiaticoside, madecassic acid and asiatic acid are the principle phytochemicals of *C. asiatica* and possess pharmacological activities such as wound healing^3,4^, memory improvement, cognition and mood modulation^5^. Although the demand for *C. asiatica* is increasing, conventional cultivation cannot guarantee phytomass and phytochemical production^6,7^. The triterpenoid amount of *C. asiatica* cultures varies among seasons, environmental conditions, cultivation regions and genotypes^1,8^.

*In vitro* hairy root culture, a disease caused by *Agrobacterium rhizogenes* infection, is an innovative platform for phytochemical production and a sustainable and economically feasible alternative to propagated plants^9^. The application of the elicitor, i.e., Methyl jasmonate (MeJA), is considered effective for enhancing terpenoid production in different plant cell, tissue and organ cultures, such as *Panax ginseng* cell culture^10^, *P. ginseng* hairy root culture^11^, *Glycyrrhiza glabra* cell culture^12^, and *C. asiatica* whole plant culture^13,14^. In addition, several studies have reported high phytomass and phytochemical production by tetraploid medicinal plants relative to the levels in normal diploid plants, such as *Papaver somniferum*^15^, *Artemisia annua*^16^ and *Salvia miltiorrhiza*^17^. Higher phytomass and triterpenoid production from tetraploid greenhouse-grown and field-grown *C. asiatica* plants have been reported^18,19^. Therefore, the aims of the present study were (1) to establish an efficient hairy root induction protocol for diploid and tetraploid *C. asiatica* and (2) to study the effects of MeJA on the phytomass and triterpenoid production of diploid and tetraploid *C. asiatica* hairy roots.

## Materials and Methods

### Plant materials

**Figure 1:**
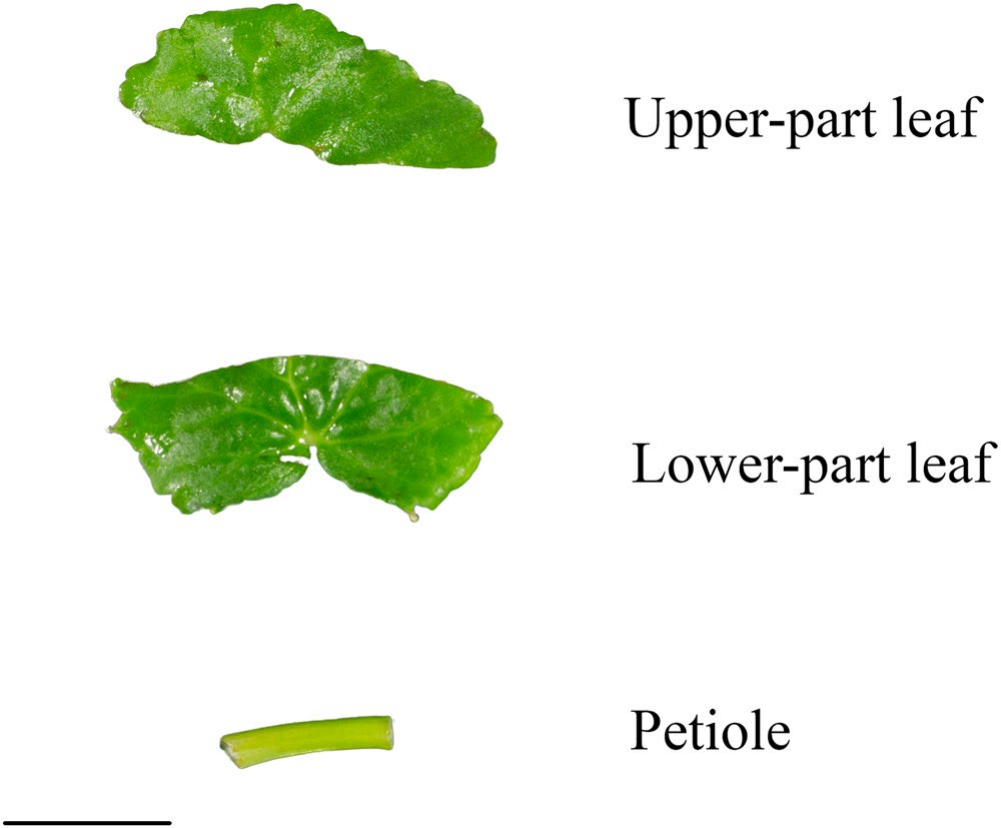
Explant types of *C. asiatica* for *A. rhizogenes* infection. Bar = 1.0 cm. (**a**) upper-part leaf explant, (**b**) lower-part leaf explant, (**c**) petiole explant.

Diploid and tetraploid *C. asiatica* plantlets were obtained from a previous study by Kaensaksiri *et al*.^18^. The plantlets were subcultured at 30-day intervals in semisolid Murashige and Skoog media (MS media)^20^ supplemented with 3.0% sucrose and containing 5.5 g/l Agargel^®^, with a pH of 5.8. The plantlets were cultured at 25 °C under a 16 h light/8 h dark photoperiod. The 24-day-old *in vitro* diploid and tetraploid *C. asiatica* plantlets were separated into petioles and upper and lower parts of leaves (Fig. 1).

### Hairy root culture induction

The hairy root induction of *C. asiatica* followed the protocol of Kim *et al*.^21^ with some modifications. One loop of each of the *A. rhizogenes* strains ATCC 43057 and ATCC 15834 (ATCC-USA) was cultured separately in 20 ml Yeast mannitol broth (YMB) medium for 3 days under a 16 h light/8 h dark photoperiod at 25 °C and agitated at 110 rpm. Three-day-old bacterial suspension cultures were used for infection.

The explants were immersed in the bacterial suspension for 40 min, placed in half-strength semisolid MS media supplemented with 50 µM acetosyringone and cultured at 25 °C in darkness. The coculture period spanned 3 days and was followed by decontamination of the bacteria with 500 mg/l cefotaxime for 14 days. The concentration of cefotaxime was reduced to 250 mg/l for the next 14 days and maintained in subculture in the same type of media until no bacteria were present.

The hairy roots were cultured in 50 ml half-strength liquid MS media, subcultured at 30-day intervals at 25 °C in the dark and agitated at 110 rpm.

### Hairy root confirmation and ploidy determination

Total DNA of the putative hairy roots was extracted by using a DNeasy plant mini kit (Qiagen, Germany) following the manufacturer’s instructions. The primers for *rolB* amplification and the Polymerase chain reaction (PCR) conditions followed those in Furner *et al*.^22^: The primers were *rolB*-1 primer 5′-GCTCTTGCAGTGCTAGATTT-3′ and *rolB*-2 primer 5′-GAAGGTGCAAGCTACCTCTC-3′; and the PCR conditions were initial denaturation at 95 °C for 2 min, 30 cycles of denaturation at 95 °C for 30 min, annealing at 53.5 °C for 45 min and extension at 72 °C for 45 min, and a final extension at 72 °C for 6 min. The PCR products were transferred to a 1.2% (w/v) agarose gel for evaluation by gel electrophoresis. The data were recorded by a Gel Documentation instrument (Gene Genius, Syngene) under UV at 260 nm after staining with SYBR^®^ Safe DNA gel stain (Fisher).

Ploidy-level determination followed the protocol of Dolezel *et al*.^23^. The data were recorded by a BD FACSCanto^TM^ flow cytometer (BD Biosciences). Diploid hairy roots were used as a control group.

### Elicitor treatments

Kim *et al*.^13^ demonstrated that asiaticoside content increased when a whole plant culture was elicited by 10–100 µM MeJA and that the optimal concentration for elicitation was 100 µM MeJA. However, they reported a negative effect of MeJA at high concentration on senescence of the whole plants. Mangas *et al*.^24^ reported that 50 µM MeJA did not elicit a whole plant culture, in contrast to the results of Kim *et al*.^13^, and that asiaticoside content increased with increasing MeJA concentration (up to 100 µM). Moreover, 200 µM MeJA treatment produced symptoms of root necrosis; thus, 100 µM MeJA was considered the optimum treatment concentration^24^. In this study, we studied the effects of MeJA on hairy root culture; as hairy root culture is more sensitive than whole plant culture, we selected a safe range of MeJA concentrations and predicted that these concentrations would produce effects. The selected concentrations of MeJA were 0 µM (as control), 50 µM and 100 µM (treatment).

A MeJA stock (Sigma-Aldrich) solution was diluted to different concentrations (i.e., 0.0 µM, 50 µM and 100 µM) by absolute ethanol (analytical grade). MeJA was applied to the hairy root cultures at the beginning of the experiment. Fresh weight (FW), Dry weight (DW) and triterpenoid production was recorded at 7-day intervals until day 28. The cultures of all treatments were cultured in the dark at 25 °C and agitated at 110 rpm.

### Growth determination and triterpenoid analysis

Three flasks of hairy roots were harvested from each MeJA treatment. FW was measured after the roots were media absorbed by sterilized tissue paper. The harvested hairy roots were dried at 40 °C for 48 h in a hot-air oven to determine DW. The hairy roots were homogenized to powder and extracted twice with 80% methanol (extraction ratio: 5:1 w/v) by sonication for 15 min. The extract was analyzed by a Thermo Scientific Ultra high-performance liquid chromatography (UHPLC) model Dionex Ultimate 3000 with a diode array detector using a protocol developed and validated by our research group (Thong-On, *et al*.^19^). A LiChroCART^®^ column (LiChrospher^®^ 100 RP-18, 250 mm × 4.0 mm I.D., particle size: 5.0 µm) and an acetonitrile and water (containing 0.1% H_3_PO_4_) gradient system were used. The mobile phase system of acetonitrile was as follows: 20–35% (10 min), 35–65% (15 min), 65–80% (5 min), 80–20% (5 min) and 20% (10 min). The flow rate was 1.0 ml/min, the injection volume was 20 µl, and 206 nm was used to detect the 4 major phytochemicals.

## Results and Discussion

### Confirmation of hairy root transformation and ploidy determination

**Figure 2:**
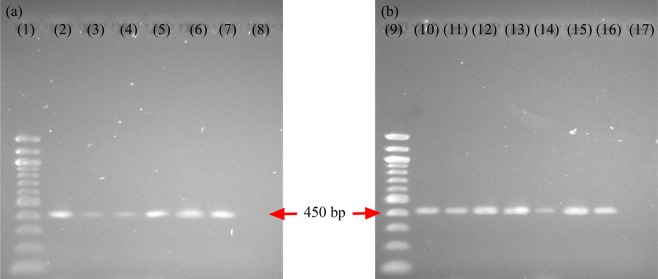
PCR analysis of the *rolB* gene of *A. rhizogenes*. (**a**) diploid hairy root lines; (**b**) tetraploid hairy root lines, lanes 1 and 9 DNA marker; lanes 8 and 17 distilled water (negative control); lanes 2–5 (HRD1, HRD2, HRD3, HRD4) and 10–14 (HRT1, HRT2, HRT3, HRT4, HRT5) amplified bands of *rolB* from the DNA of hairy root lines; lanes 6–7 and 15–16 amplified bands of *rolB* from *A. rhizogenes* (positive control).

The transgenic state of the hairy root lines was confirmed by the presence of *rolB* in the putative hairy root genome. There were 4 diploid hairy root lines (HRD1–4) (Fig. 2a) and 5 tetraploid hairy root lines (HRT1-5) (Fig. 2b). The healthy diploid and tetraploid hairy root lines were chosen to conduct the elicitation experiment.

**Figure 3:**
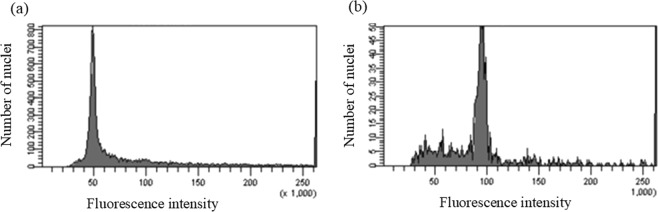
Flow cytometry histogram of *C. asiatica* hairy roots. (**a**) Histogram of a diploid control hairy root line, (**b**) histogram of a tetraploid hairy root line.

In addition, *rolB* was detected in these hairy root lines, confirming the transformation (Supplement Information). The ploidy levels of the hairy root lines obtained from the tetraploid explants were determined by flow cytometry. The peaks of the tetraploid hairy roots (4x) and diploid control hairy roots (2x) were set at the channels 100 and 50, respectively (Fig. 3).

### Effects of explant type and *A. rhizogenes* strain on hairy root induction

At both ploidy levels, hairy roots were induced from the lower part of the leaf explants after 15–17 days, whereas 29 days elapsed before the petiole explants produced hairy roots. There was no hairy root induction from the upper parts of the leaves of explants of either ploidy level of *C. asiatica*. Hairy roots were induced directly from the cut ends of the explants. The hairy roots induced from tetraploid explants were confirmed to be tetraploid by flow cytometry. A higher transformation rate was observed from the lower parts of the leaves of diploid (5–10%) and tetraploid explants (4.17–7.14%) (Table 1). The *A. rhizogenes* strain ATCC 43057 was more virulent than the strain ATCC 15834 and thus infected more explant types, i.e., the petioles and lower parts of leaves, and induced a higher transformation rate. Variation in *A. rhizogenes* strain and explant type can lead to different results because of differences among bacteria in the ability to induce hairy roots and the different responses of explants to bacteria^25,26^. The high transformation rate of the lower parts of the leaves may have been observed because of the larger midveins at the base of the leaves than in the upper parts; these might have allowed a large number of bacterial host cells to be integrated into the genome of the plant. Moreover, the lower parts of the leaves had 2 cut ends, which increased the infection area relative to that at the upper parts. Therefore, the use of the lower parts of the leaves for infection by *A. rhizogenes* strain ATCC 43057 for three days in the darkness was efficient for achieving a high successful transformation rate for *C. asiatica* hairy root induction.

**Table 1:** Effect of explant type and *A. rhizogenes* strain on hairy root induction (%) of *C. asiatica*.

Ploidy level	Explant types	*A. rhizogenes* strain		
		Control	ATCC 43057	ATCC 15834
Diploid plant	Upper-leaf	—	—	—
	Lower-leaf	—	10.00%	5.00%
	Petiole	—	2.38%	—
Tetraploid plant	Upper-leaf	—	—	—
	Lower-leaf	—	7.14%	4.17%
	Petiole	—	5.26%	—

**Figure 4:**
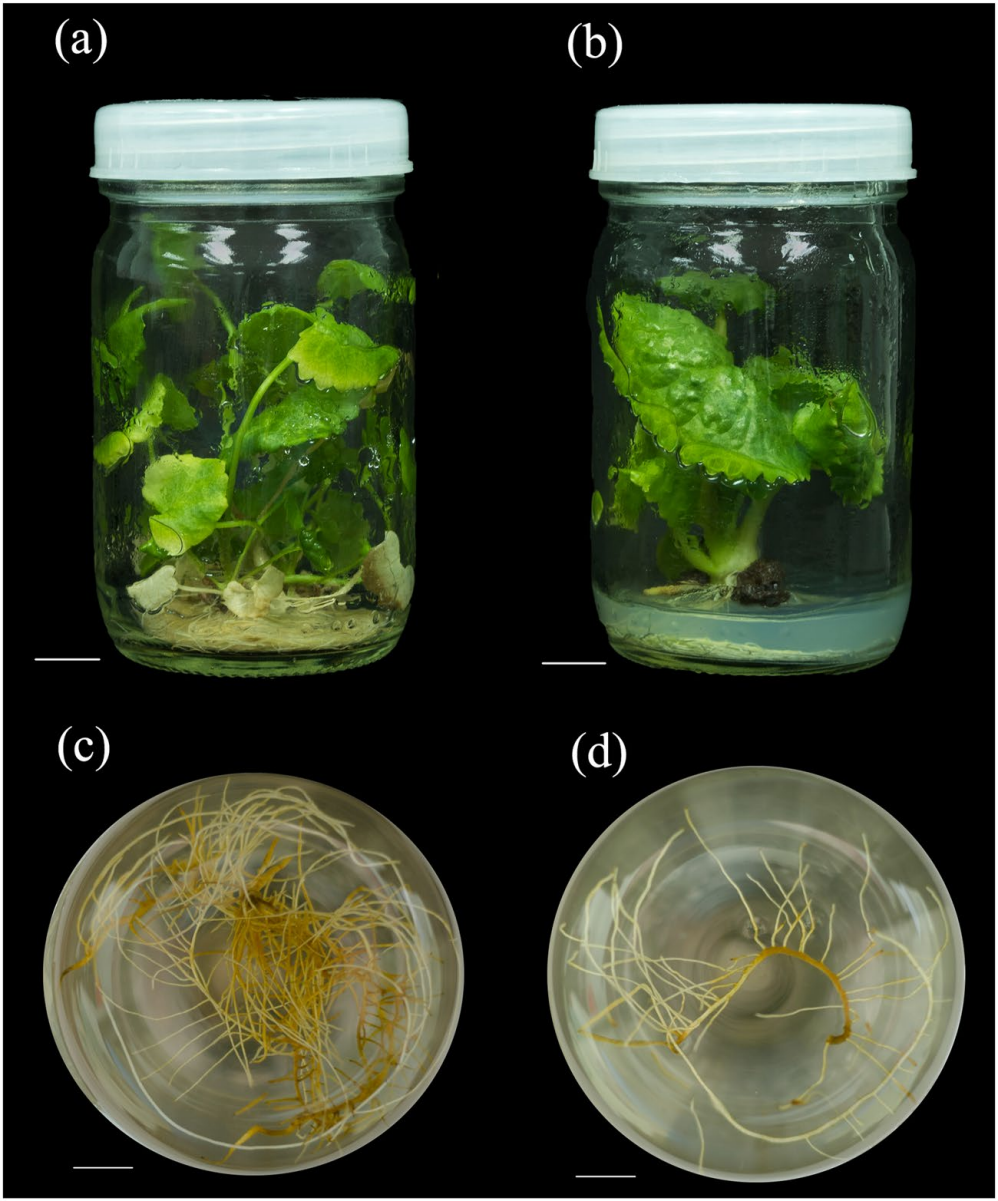
Diploid (**a**) and tetraploid (**b**) plants and derived diploid (**c**) and tetraploid (**d**) hairy root cultures of *C. asiatica*. Bar = 1.0 cm.

The characteristics of the hairy roots of differed between the diploid and tetraploid *C. asiatica* explants (Fig. 4). The diploid hairy roots displayed high lateral branch formation and rapid growth. Moreover, the dimensions of the primary hairy roots were similar to those of the lateral branches. However, the tetraploid hairy roots grew slowly and produced fewer lateral roots. The dimensions of the primary roots were larger than those of the lateral branches in tetraploid *C. asiatica* hairy roots.

### Effects of MeJA on the growth of hairy root cultures

**Figure 5:**
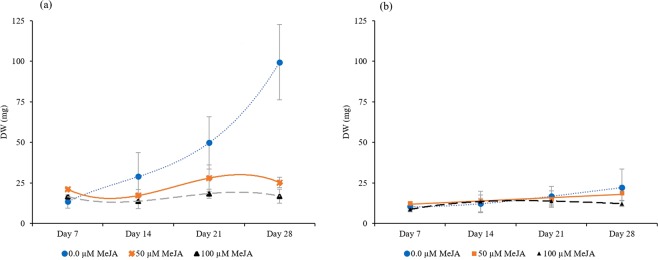
Growth patterns of diploid (**a**) and tetraploid (**b**) *C. asiatica* hairy root cultures. The data are presented as mean ± SD.

Figure 5 demonstrates the growth patterns of diploid and tetraploid hairy root cultures treated with different MeJA treatments. For the diploid hairy root cultures, the control treatment produced higher DW at day 28 than the other MeJA treatments, reaching 99.44 ± 23.2 mg/flask. The hairy root cultures of the 50 µM MeJA treatment and the 100 µM MeJA treatment showed maximum DW of 28.02 ± 8.05 mg/flask and 18.50 ± 2.78 mg/flask, respectively, on day 21. MeJA inhibited the growth of diploid hairy roots, especially those treated with high MeJA concentrations. The maximum DW for the tetraploid hairy root cultures was 22.14 ± 11.48 mg/flask at day 28 for the control treatment, 18.86 ± 4.58 mg/flask at day 28 for the 50 µM MeJA treatment and 13.88 ± 2.34 mg/flask at day 21 for the 100 µM MeJA treatment. The nonelicited tetraploid hairy roots grew but at a slower growth rate than the nonelicited diploid hairy roots. The growth of the nonelicited diploid hairy roots was 4.5-fold higher than that of nonelicited tetraploid hairy roots at day 28. Inhibitory effects of MeJA have previously been reported on hairy root cultures of *Rhinacanthus nasutus*^27^ and *Glycine max*^28^. The reasons may be related to the phenolic compounds secreted from hairy roots, which are stimulated by the stress from MeJA, or the toxic effect of MeJA itself on the hairy roots^29^. Moreover, polyploidy plants may have a small number of cell divisions during growth and development that result in a lower growth rate than in normal diploid plants^30,31^. De Jesus-Gonzalez and Weathers^32^ reported similar results for *Artemisia annua*.

### Effects of MeJA on the triterpenoid production of hairy root cultures

There was no triterpenoid production in nonelicited *C. asiatica* hairy root culture, whereas MeJA led to triterpenoid production in both diploid and tetraploid hairy root cultures (Tables 2 and 3). For the diploid hairy root cultures, the highest triterpenoid production under the 50 and 100 µM MeJA treatments was 27.25 ± 0.27 µg/mg DW at day 21 and 6.17 ± 2.30 µg/mg DW at day 14, respectively. When treated on the same day, the 50 µM MeJA treatment produced more triterpenoids than the 100 µM MeJA treatment. For the tetraploid hairy root cultures, in the 50 µM MeJA treatment, the hairy root culture at day 28 produced the maximum amount of triterpenoids, 16.29 ± 6.32 µg/mg DW, whereas the 100 µM MeJA treatment produced a similar triterpenoid amount (16.31 ± 9.24 µg/mg DW) at day 14 (Tables 2 and 3). Ploidy level (*p* = 0.0334), MeJA treatment (*p* = 0.0000) and number of days of culture (*p* = 0.0003) were the main factors that significantly affected the total triterpenoid production of *C. asiatica* hairy roots, as determined by 3-way analysis of variance. Furthermore, the triterpenoid production patterns in each ploidy level for all MeJA treatments differed in their production characteristics. Asiaticoside was dominant, followed by asiatic acid and madecassic acid. Madecassoside was not detected in hairy roots of either ploidy level. Interestingly, there were different ratios of aglycones (i.e., asiatic acid and madecassic acid) to glycoside (i.e., asiaticoside) when the hairy roots were treated with MeJA. The ratios in diploid and tetraploid hairy roots treated with 50 µM MeJA at day 28 were 1:7 and 1:2, respectively. However, the ratios under 100 µM MeJA treatment were similar between the diploid and tetraploid hairy roots, e.g., 1:1 on day 28. These results showed that the ratio of aglycones to glycoside in diploid hairy roots was affected by the concentration of MeJA, whereas the ratio was largely stable in the tetraploid hairy roots, even at high MeJA concentrations. Aziz *et al*.^33^ and Kim *et al*.^34^ reported that there was no triterpenoid production from nonelicited *C. asiatica* hairy root cultures. This result may be related to organ-specific biosynthesis, which involves interactions between plant organs and plant metabolic precursors produced in roots and transported to aerial parts for bioconversion^33^. Moreover, the genes associated with triterpenoid biosynthesis may be poorly expressed in transgenic roots^34^. In contrast, the addition of MeJA to the hairy root cultures resulted in triterpenoid production, possibly because of the upregulation of *CaSQS* (*Centella asiatica* squalene synthase) and *CabAS* (*Centella asiatica β*-amyrin synthase) genes^21,35^. Previous studies reported different proportions of triterpenoids in 4-month-old field-grown diploid and tetraploid *C. asiatica* plants^18,19^. The ratios of aglycones to glycosides were 1:13 and 1:9 in diploid and tetraploid leaves, respectively. In the present study, the tetraploid hairy roots produced more glycones than did the diploid hairy roots, which increased the aglycone/glycoside ratio to 1:2 and 1:1 under treatment with 50 and 100 µM MeJA, respectively, at day 28. The downregulation of UDP-glucosyltransferases in the tetraploid hairy roots by MeJA may have contributed to the different proportions between the plants of different ploidies^36^. Commercial drugs produced from *C. asiatica* and other preparations consist of 60% aglycones and 40% asiaticoside (equivalent to a 3:2 ratio)^1,2^. The manufacturing processes of these *C. asiatica* drugs and preparations are complex and involve chemical treatments that cannot maintain the proportions of natural components. Purified chemicals are also added to enrich the extract, which may affect the price of *C. asiatica* products^2^. By using elicited tetraploid hairy roots, these production obstacles may be solved. The findings indicate that *C. asiatica* hairy roots cannot produce the four major triterpenoids without intervention, which may be related to organ-specific biosynthesis. The application of MeJA resulted in not only triterpenoid production but also differences in triterpenoid proportion between diploid and tetraploid hairy root cultures. The diploid hairy roots were suitable for glycoside production, and the tetraploid hairy roots were suitable for aglycone production. Moreover, 50 µM MeJA was the optimum concentration for maximizing the amount of triterpenoids per flask from *C. asiatica* hairy roots of both ploidy levels.

**Table 2:** Triterpenoid Production of diploid hairy root cultures (Mean ± SD (µg/mg DW)).

MeJA	Harvest Date	AD	MA	AA	TT
0 µM	Day 7	0.00 ± 0.00^a^	0.00 ± 0.00^a^	0.00 ± 0.00^a^	0.00 ± 0.00^a^
	Day 14	0.00 ± 0.00^a^	0.00 ± 0.00^a^	0.00 ± 0.00^a^	0.00 ± 0.00^a^
	Day 21	0.00 ± 0.00^a^	0.00 ± 0.00^a^	0.00 ± 0.00^a^	0.00 ± 0.00^a^
	Day 28	0.00 ± 0.00^a^	0.00 ± 0.00^a^	0.00 ± 0.00^a^	0.00 ± 0.00^a^
50 µM	Day 7	15.35 ± 9.71^bc^	0.00 ± 0.00^a^	0.00 ± 0.00^a^	15.35 ± 9.71^c^
	Day 14	11.67 ± 3.49^b^	0.00 ± 0.00^a^	0.00 ± 0.00^a^	11.67 ± 3.49^bc^
	Day 21	25.87 ± 0.93^d^	0.47 ± 0.16^bc^	0.91 ± 0.53^abc^	27.25 ± 0.27^d^
	Day 28	18.82 ± 5.90^c^	0.79 ± 0.50^c^	2.24 ± 0.92^d^	22.85 ± 7.32^d^
100 µM	Day 7	4.20 ± 0.76^a^	0.00 ± 0.00^a^	0.00 ± 0.00^a^	4.20 ± 0.76^a^
	Day 14	5.20 ± 2.10^a^	0.35 ± 0.15^ab^	0.62 ± 0.33^ab^	6.17 ± 2.30^ab^
	Day 21	3.04 ± 1.60^a^	0.44 ± 0.50^bc^	1.28 ± 1.43^bc^	4.76 ± 3.44^ab^
	Day 28	2.76 ± 0.63^a^	0.55 ± 0.11^bc^	1.66 ± 0.30 ^cd^	4.96 ± 1.03^ab^

Means within each column with different letters are significantly different at P < 0.05.

TT: Total triterpenoid, AD: Asiaticoside, MA: Madecassic Acid, AA: Asiatic Acid.

**Table 3:** Triterpenoid Production of tetraploid hairy root cultures (Mean ± SD (µg/mg DW)).

MeJA	Harvest Date	AD	MA	AA	TT
0 µM	Day 7	0.00 ± 0.00^a^	0.00 ± 0.00^a^	0.00 ± 0.00^a^	0.00 ± 0.00^a^
	Day 14	0.00 ± 0.00^a^	0.00 ± 0.00^a^	0.00 ± 0.00^a^	0.00 ± 0.00^a^
	Day 21	0.00 ± 0.00^a^	0.00 ± 0.00^a^	0.00 ± 0.00^a^	0.00 ± 0.00^a^
	Day 28	0.00 ± 0.00^a^	0.00 ± 0.00^a^	0.00 ± 0.00^a^	0.00 ± 0.00^a^
50 µM	Day 7	0.00 ± 0.00^a^	0.00 ± 0.00^a^	0.00 ± 0.00^a^	0.00 ± 0.00^a^
	Day 14	0.00 ± 0.00^a^	0.37 ± 0.20^ab^	0.00 ± 0.00^a^	0.37 ± 0.20^a^
	Day 21	5.83 ± 3.37^abc^	0.65 ± 0.23^ab^	1.84 ± 0.29^ab^	8.32 ± 2.87^abc^
	Day 28	10.72 ± 4.25^cd^	1.40 ± 0.69^cd^	4.17 ± 1.38^c^	16.29 ± 6.32^c^
100 µM	Day 7	3.49 ± 3.03^ab^	0.00 ± 0.00^a^	0.00 ± 0.00^a^	3.49 ± 3.03^ab^
	Day 14	14.39 ± 7.86^d^	0.51 ± 0.21^ab^	1.41 ± 1.22^ab^	16.31 ± 9.24^c^
	Day 21	7.60 ± 7.19^bc^	0.82 ± 0.58^bc^	2.20 ± 1.9^b^	10.62 ± 9.34^bc^
	Day 28	7.95 ± 2.06^bc^	1.85 ± 1.04^d^	5.69 ± 1.96^c^	15.49 ± 4.93^c^

Means within each column with different letters are significantly different at P < 0.05.

TT: Total triterpenoid, AD: Asiaticoside, MA: Madecassic Acid, AA: Asiatic Acid.

## Supplementary information

Dataset 1
