# Supplementary material for: Effects of methyl jasmonate on the growth and triterpenoid production of diploid and tetraploid Centella asiatica (L.) Urb. hairy root cultures
Source: Sci Rep. 2019 Dec 10;9:18665. doi: 10.1038/s41598-019-54460-z (PMC6904556; doi:10.1038/s41598-019-54460-z)
Supplement: Supplementary file 1 — Dataset 1 [file 41598_2019_54460_MOESM1_ESM.docx]

**Effects of methyl jasmonate on the growth and triterpenoid production of diploid and tetraploid *Centella asiatica* (L.) Urb. hairy root cultures**

Khoa Van Nguyen, Benyakan Pongkitwitoon, Thanika Pathomwichaiwat, Unchera Viboonjun, Sompop Prathanturarug^[[1]](#footnote-1)^

**Supplementary information**

When we run the gel electrophoresis, we put all the genes of diploid hairy root lines in 1 gel, and the tetraploid hairy root lines’ in another gel. So, I had 2 different gels contained 2 different ploidy levels. In the figure 2, we cropped the wells of gels and the exposure was made by Gel Documentation machine (Gene Genius, Syngene). We include here more photos from Gel Doc machine with different exposures, negative color and 3D - image (Fig. Supp. 1).

**Fig. Supp. 1: PCR analysis of *rolB* gene of *A. rhizogenes* with different exposures, negative color and 3D - image**

**(a) diploid hairy root lines**

1 DNA marker; 8 distilled water (negative control); 2-5 (HRD1, HRD2, HRD3, HRD4) amplified bands of *rolB* from the DNA of hairy root lines; 6-7 amplified bands of *rolB* from *A. rhizogenes* (positive control)


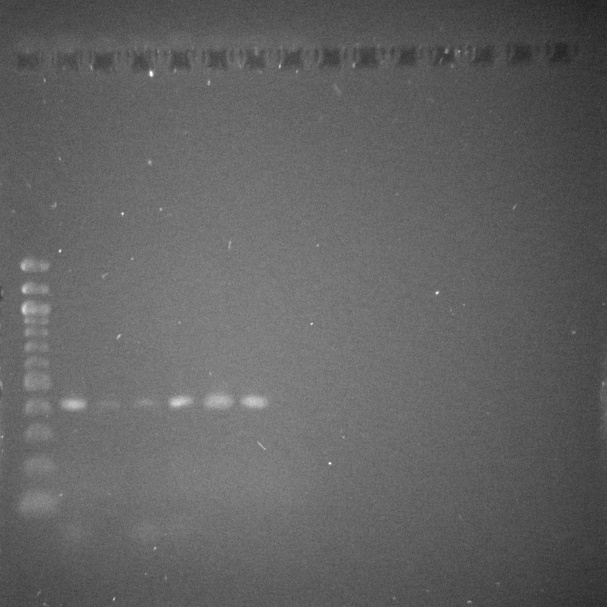


(a)

(1) (2) (3) (4) (5) (6) (7) (8)

🡸 450 bp


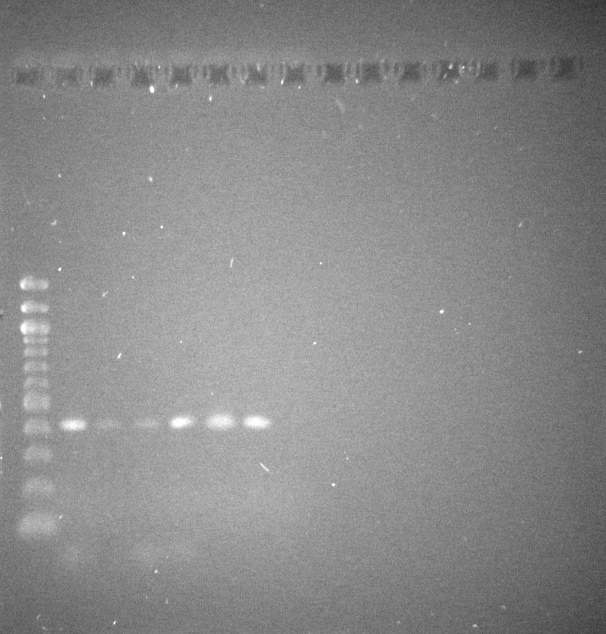


(a)

(1) (2) (3) (4) (5) (6) (7) (8)

🡸 450 bp


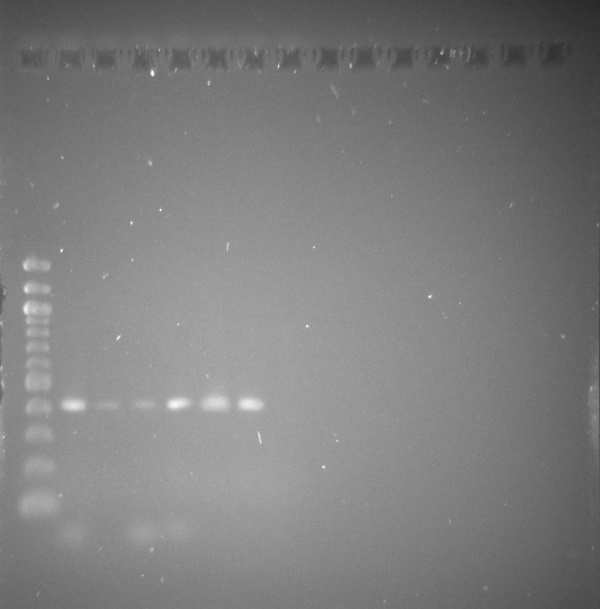


(a)

(1) (2) (3) (4) (5) (6) (7) (8)

🡸 450 bp


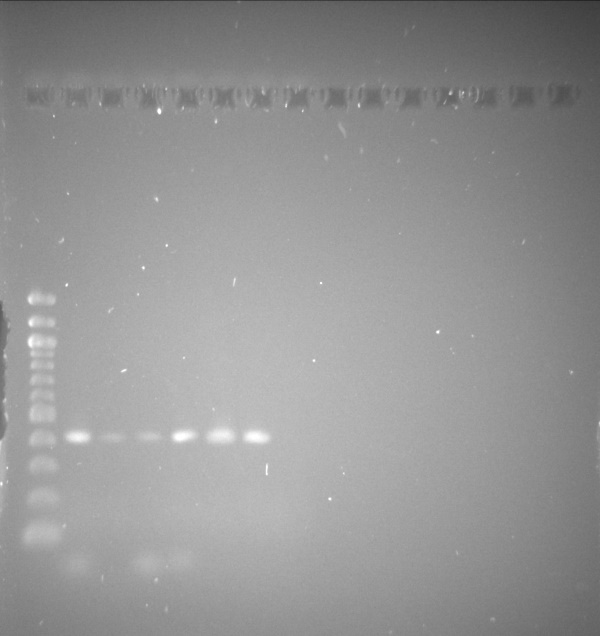


(a)

(1) (2) (3) (4) (5) (6) (7) (8)

🡸 450 bp


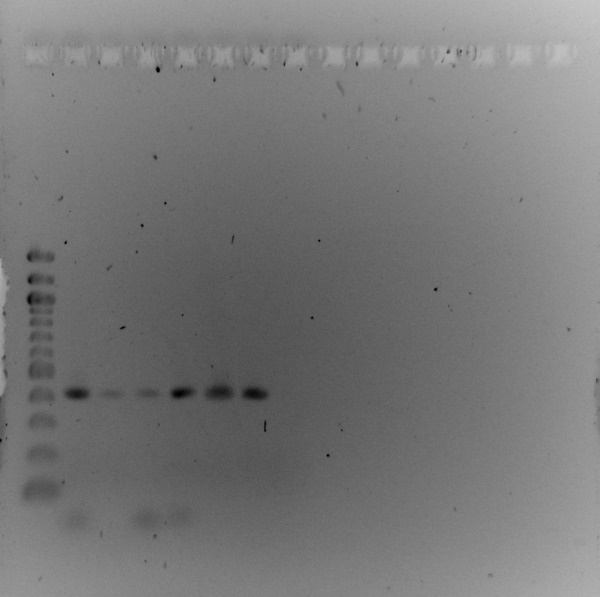


(a)

(1) (2) (3) (4) (5) (6) (7) (8)

Negative color

🡸 450 bp


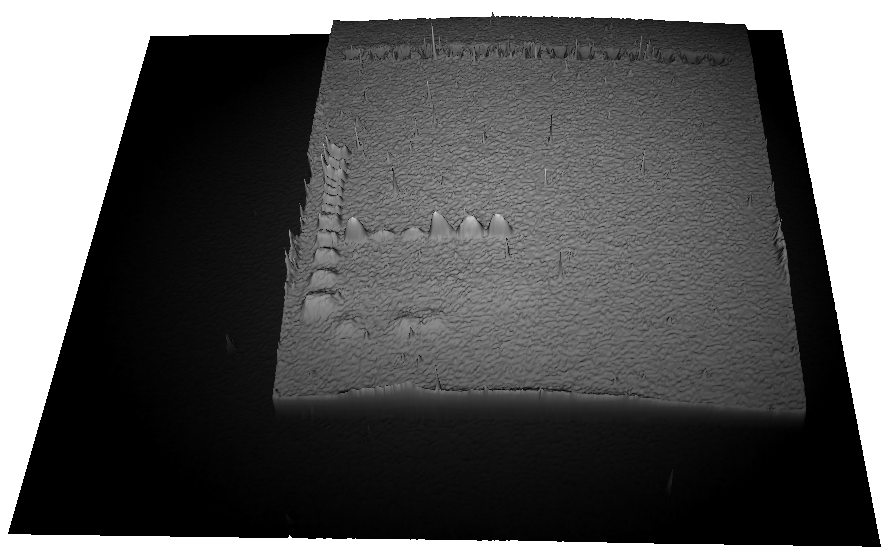


(a)

(1) (2)(3)(4) (5)(6)(7)(8)

3D - Image

🡸 450 bp

**(b) tetraploid hairy root lines**

9 DNA marker; 17 distilled water (negative control); 10-14 (HRT1, HRT2, HRT3, HRT4, HRT5) amplified bands of *rolB* from the DNA of hairy root lines; 15-16 amplified bands of *rolB* from *A. rhizogenes* (positive control)


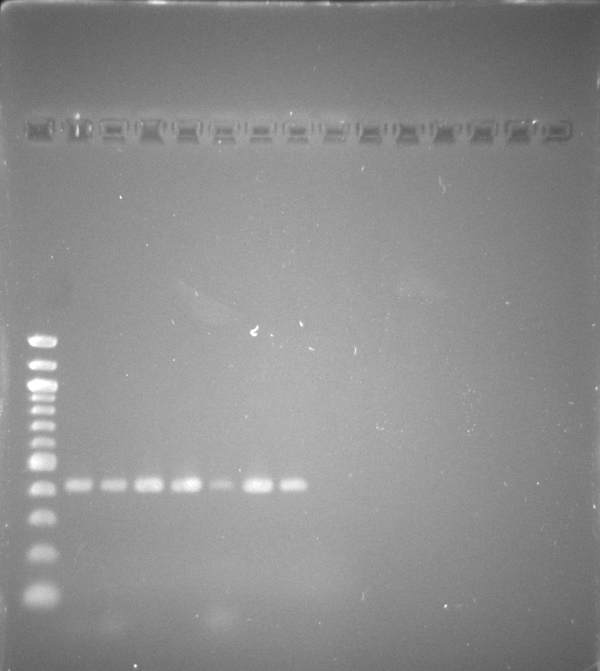


(b)

(9) (10)(11)(12)(13)(14)(15)(16)(17)

🡸 450 bp


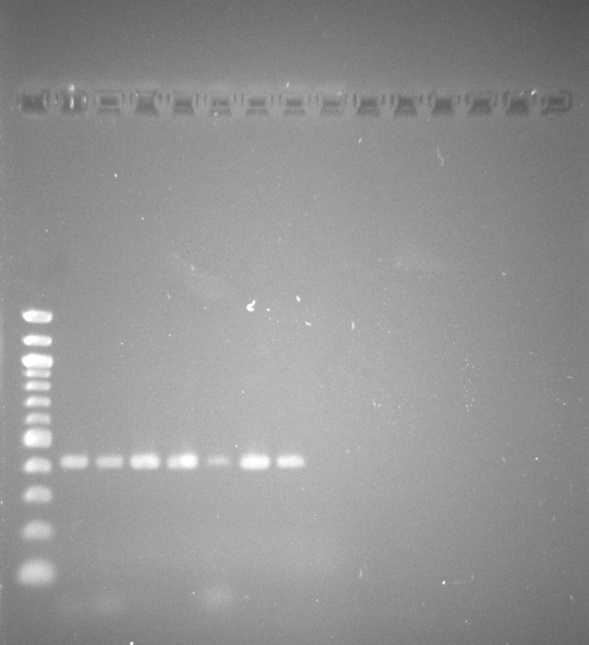


(b)

(9) (10)(11)(12)(13)(14)(15)(16)(17)

🡸 450 bp


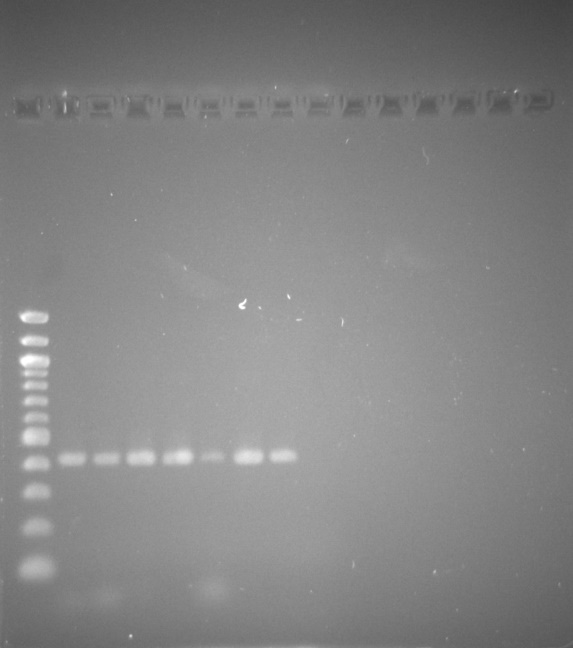


(b)

(9) (10)(11)(12)(13)(14) (15)(16)(17)

🡸 450 bp


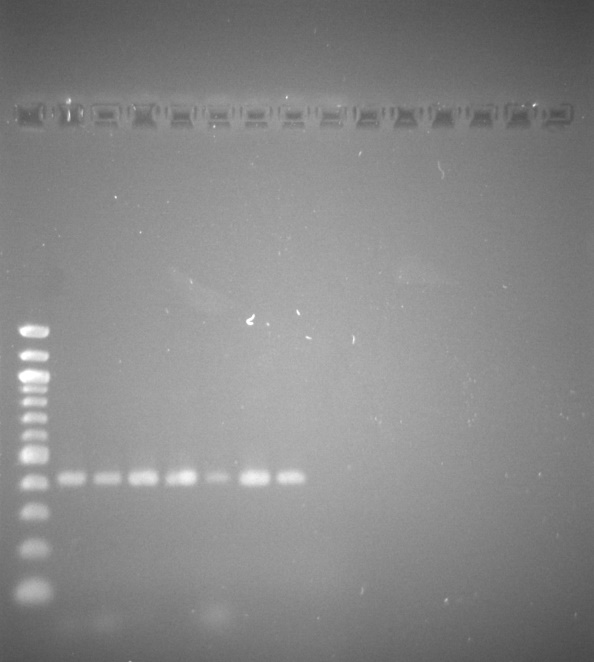


(b)

(9) (10)(11)(12)(13)(14)(15)(16)(17)

🡸 450 bp


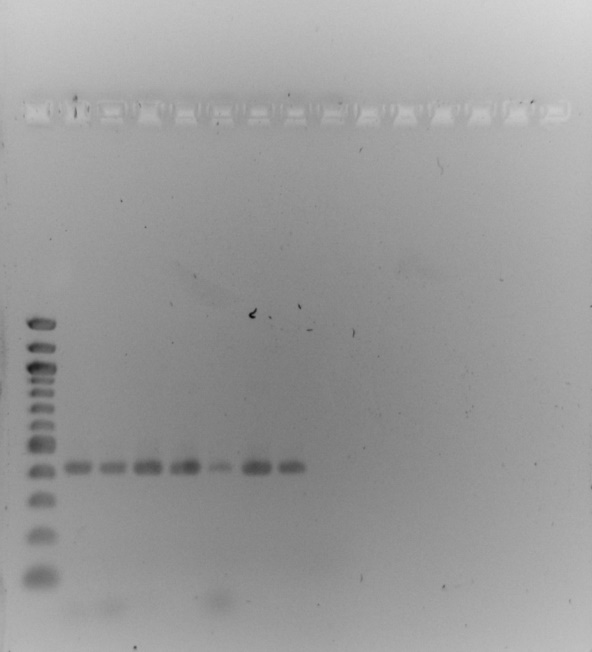


(b)

(9) (10)(11)(12) (13)(14)(15)(16)(17)

🡸 450 bp

Negative color


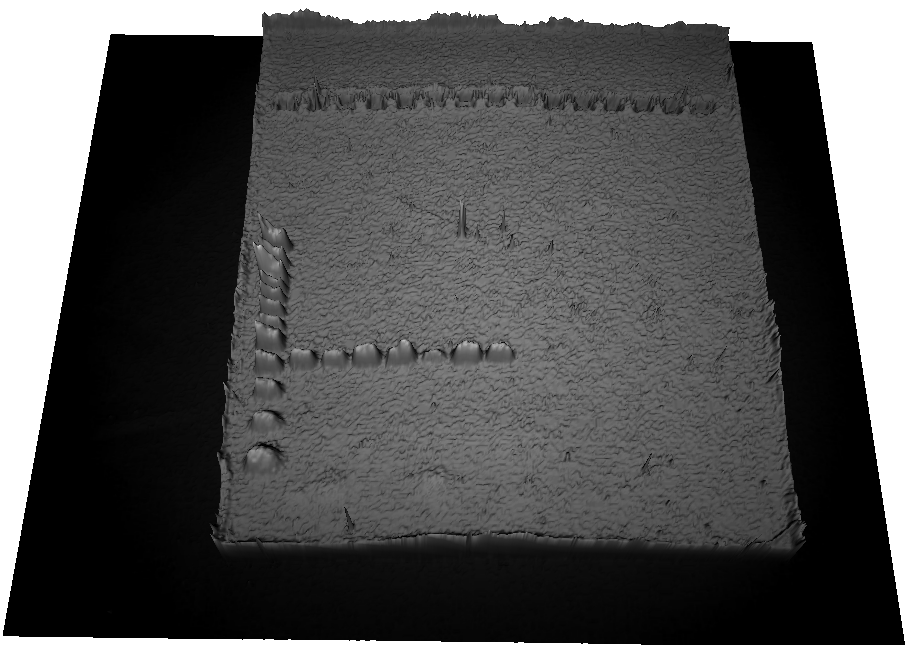


(b)

(9)(10)(11)(12)(13)(14)(15)(16)(17)

🡸 450 bp

3D - Image

1. [↑](#footnote-ref-1)
